# New insights into the evolution of the *Trypanosoma cruzi* clade provided by a new trypanosome species tightly linked to Neotropical *Pteronotus* bats and related to an Australian lineage of trypanosomes

**DOI:** 10.1186/s13071-015-1255-x

**Published:** 2015-12-23

**Authors:** Luciana Lima, Oneida Espinosa-Álvarez, C. Miguel Pinto, Manzelio Cavazzana Jr., Ana Carolina Pavan, Julio C. Carranza, Burton K. Lim, Marta Campaner, Carmen S. A. Takata, Erney P. Camargo, Patrick B. Hamilton, Marta M. G. Teixeira

**Affiliations:** Departamento de Parasitologia, Instituto de Ciências Biomédicas, Universidade de São Paulo, Av. Lineu Prestes, 1374, 05508-000 São Paulo, SP Brazil; Division of Mammals, National Museum of Natural History, Smithsonian Institution, Washington, DC USA; Centro de Investigación en Enfermedades Infecciosas y Crónicas, Escuela de Biología, Pontificia Universidad Católica del Ecuador, Quito, Ecuador; Faculdades Integradas Padre Albino (FIPA) e Faculdade de Ciências da Saúde de Barretos (FACISB), Barretos, SP Brazil; Departamento de Biologia, Instituto de Biociências, Universidade de São Paulo, São Paulo, SP Brazil; Laboratorio de Investigaciones en Parasitología Tropical, Universidad del Tolima, Ibagué, Colombia; Department of Natural History, Royal Ontario Museum, Toronto, Canada; Biosciences, College of Life and Environmental Sciences, University of Exeter, Exeter, UK

**Keywords:** Chiroptera, Bat trypanosomes, Museum archives, Phylogeny, Evolution, Phylogeography, Australia, Neotropics, Host-parasite association

## Abstract

**Background:**

Bat trypanosomes are implicated in the evolution of the *T. cruzi* clade, which harbours most African, European and American trypanosomes from bats and other trypanosomes from African, Australian and American terrestrial mammals, including *T. cruzi* and *T. rangeli,* the agents of the American human trypanosomiasis*.* The diversity of bat trypanosomes globally is still poorly understood, and the common ancestor, geographical origin, and evolution of species within the *T. cruzi* clade remain largely unresolved.

**Methods:**

Trypanosome sequences were obtained from cultured parasites and from museum archived liver/blood samples of bats captured from Guatemala (Central America) to the Brazilian Atlantic Coast. Phylogenies were inferred using Small Subunit (SSU) rRNA, glycosomal glyceraldehyde phosphate dehydrogenase (gGAPDH), and Spliced Leader (SL) RNA genes.

**Results:**

Here, we described *Trypanosoma wauwau* n. sp. from *Pteronotus* bats (Mormoopidae) placed in the *T. cruzi* clade, then supporting the bat-seeding hypothesis whereby the common ancestor of this clade likely was a bat trypanosome. *T. wauwau* was sister to the clade *T.* spp-Neobats from phyllostomid bats forming an assemblage of trypanosome species exclusively of Noctilionoidea Neotropical bats, which was sister to an Australian clade of trypanosomes from indigenous marsupials and rodents, which possibly evolved from a bat trypanosome. *T. wauwau* was found in 26.5 % of the *Pteronotus* bats examined, and phylogeographical analysis evidenced the wide geographical range of this species*.* To date, this species was not detected in other bats, including those that were sympatric or shared shelters with *Pteronotus. T. wauwau* did not develop within mammalian cells, and was not infective to Balb/c mice or to triatomine vectors of *T. cruzi* and *T. rangeli*.

**Conclusions:**

*Trypanosoma wauwau* n. sp. was linked to *Pteronotus* bats. The positioning of the clade *T. wauwau/T.*spp-Neobats as the most basal Neotropical bat trypanosomes and closely related to an Australian lineage of trypanosomes provides additional evidence that the *T. cruzi* clade trypanosomes likely evolved from bats, and were dispersed in bats within and between continents from ancient to unexpectedly recent times.

**Electronic supplementary material:**

The online version of this article (doi:10.1186/s13071-015-1255-x) contains supplementary material, which is available to authorized users.

## Background

The number of studies on bat trypanosomes from the New and Old Worlds that proposed that *T. cruzi* and *T. rangeli* evolved from within the broader monophyletic assemblage of the *T. cruzi* clade is increasing. This clade was formed mainly by trypanosomes of bats, and some other mammalian hosts in the Americas, Africa and Australia. Accordingly, it was proposed the bat-seeding hypothesis, in which a common ancestor bat trypanosome gave origin (speciation) to several trypanosomes that evolved linked to bats or have switched, by several independent events at different times, into a range of terrestrial mammals in the New and Old Worlds, then originating several lineages (monophyletic assemblages) of bat trypanosomes [1–5].

Regardless of their traditional taxonomic classification, morphology and development in cultures, or ranges of host species and geographical distributions, trypanosomes nested into the *T. cruzi* clade are distributed in two main sister phylogenetic lineages. One lineage represents the subgenus *Schizotrypanum* that harbours *T. cruzi,* which is a species found in bats and mammals of virtually all terrestrial orders from the southern United States to southern South America. The other species within the subgenus *Schizotrypanum* are all restricted to bats: *T. dionisii* found in bats from the New and Old Worlds, *T. cruzi marinkellei* of Central and South America, and *T. erneyi* of African bats [3, 5–9]. The second lineage (*T. rangeli*/*T. conorhini*) comprises two sister clades. One clade is exclusive of *T. rangeli* from humans, monkeys, rodents, xenarthrans, bats and other mammals. The other clade includes *T. conorhini* (tropicopolitan of rats), *T. vespertilionis* (European bats), and African trypanosomes from bats, monkeys and civets. The lineage of Australian trypanosomes from marsupials and rodents were basal to these lineages [1, 3, 4, 10].

*T. livingstonei* from African bats was placed at the edge of the *T. cruzi* clade [4]. Recently, PCR surveys revealed new trypanosome species in phyllostomid bats from Panamá positioned at the base of the clade *T. cruzi.* However, the relationships of the new trypanosomes with *T. livingstonei* and the Australian trypanosomes were unresolved [11]. In a likely evolutionary scenario, all trypanosome species within the *T. cruzi* clade evolved from an Old World bat trypanosome, possibly in Africa where the most basal species was found so far, and from where bats irradiated in the Eocene [1–4, 10]. Therefore, further surveys of the trypanosomes in bats of the New and Old Worlds are required to shed more light on the evolution of these intriguing parasites, and on the emergence of the human infective bat trypanosomes *T. cruzi* and *T. rangeli*.

The discovery of bat trypanosomes in Europe and Africa that were highly closely related to bat trypanosomes in South America suggests natural movements of bats carrying trypanosomes across continents more recently than those suggested by the fossil records [1, 2]. Apparently, the constant movements of hosts (vertebrates and invertebrates) shaped the diversity, phylogenetic relationships, ranges of vertebrate and vector species, and present day distributions of trypanosomatids in general. Phylogeographical analyses have revealed unexpected distributions of trypanosome and leishmania species across the world [1, 2, 12–14].

Surveys and molecular characterization of bat trypanosomes conducted by our and other research groups in Brazil, Panama, Colombia, Bolivia and Ecuador [2–5, 9, 11, 15–20] discovered a large repertoire of bat trypanosomes, revealing a range of genotypes of *T. cruzi, T. rangeli*, *T. dionisii* and *T. c marinkellei,* and the existence of an increasing number of trypanosomes diverging by relevant genetic distance from any known trypanosome species, including one different trypanosome species found exclusively in bats of *Pteronotus* [9].

The genera *Pteronotus* and *Mormoops* constitute the Mormoopidae family of strictly insectivorous Neotropical bats. The species of *Pteronotus* live in warm regions near water sources and form large colonies in caves and under bridges often together with phyllostomid bats [21]. This genus is currently Neotropical and, in Brazil, *Pteronotus* spp. are quite common in Amazonia and Cerrado biomes, and were recently found in the Atlantic Forest of northeastern Brazil [22]. The Mormoopidae is sister to Phyllostomidae and closely allied with Noctilionidae, Furipteridae and Natalidae, which together form the Noctilionoidea superfamily widespread in the Neotropics and comprising one extant species of Myzopodidae in Australia, and a single species of Mystacinidae in New Zealand [23–27].

In the present study, we carried out a comprehensive survey of the trypanosomes infecting *Pteronotus* bats from Central and South America. The molecular characterization of the trypanosomes revealed a link between bats of *Pteronotus* and a new species of trypanosome, which will be described in this study using a combination of phylogenetic, morphological, biological, and eco-biogeographical data.

## Methods

### Capture and identification of bats, and isolation of trypanosomes in culture

**Fig. 1:**
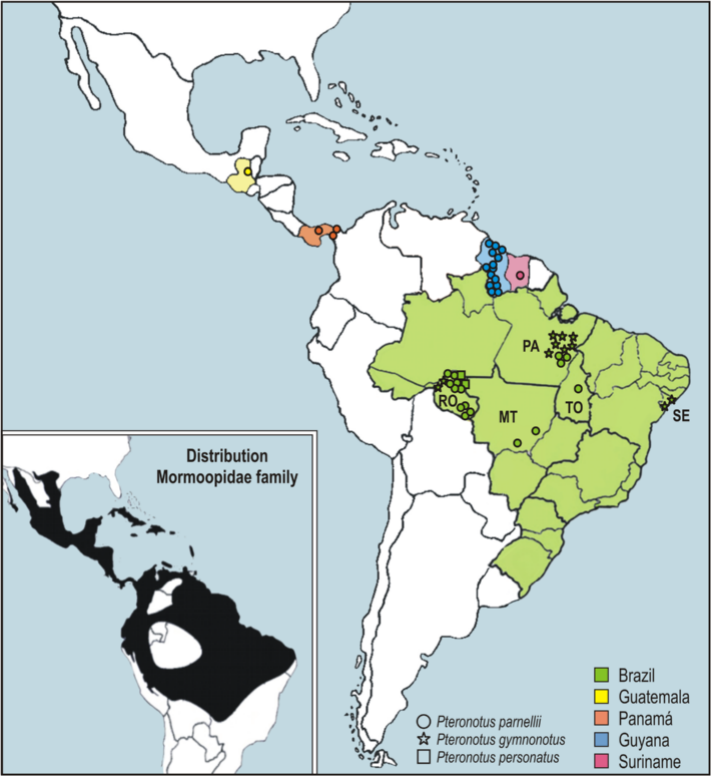
Geographical origin of *Trypanosoma wauwau* isolates obtained from hemocultures and archive blood/tissue samples from *Pteronotu*s bats captured in Central and South America

Bats of the genus *Pteronotus* were captured using mist nets in two localities in the State of Rondonia, Amazonia biome, Brazil (Fig. 1) in 2001, 2002, 2005 and 2009. The bats were anaesthetised and manipulated for blood sampling as previously described [4, 9].

#### Ethical approval

All procedures in Brazil were in accord with the Committee on the Ethics of Animal Experimentation of the Institutes of Biomedical Sciences and Biosciences, University of São Paulo (Approved protocols:n^o^17/page 3/book2 and n^o^109/03), and with the recommendations of the Brazilian Institute of the Environment and Renewable Natural Resources (IBAMA- Permit Number 10080-2). The bats from other countries were manipulated according to procedures approved by the Royal Ontario Museum (Toronto, Canada) for previous studies [24]. Bat blood samples (100–200 ul) were submitted to haemoculture (HE) as we described previously [4]. The bats captured in Brazil were identified with morphological keys and representative specimens of each species (deposited in the Zoological Museum of the University of São Paulo) were confirmed using DNA from liver/blood samples preserved in ethanol for PCR amplification and sequencing of the Cytochrome b (Cytb) and the Cytochrome c oxidase subunit I (COI) genes [28]. The barcode sequences were analysed by BLAST search in GenBank, and the bats were identified as *P. parnellii*, *P. personatus* and *P. gymnonotus.* A study is currently being developed using these sequences aiming the taxonomic revision of the genus *Pteronotus* (Pavan et al., manuscript in preparation).

### Archived blood samples from bats of the genus *Pteronotus*

We tested 101 DNA samples from archived tissue (liver) samples from *Pteronotus* spp. captured in four biomes: Amazonia (States of Pará and Mato Grosso); transitional areas between Amazonia and Cerrado (Maranhão); Cerrado (Goias, Mato Grosso, Piauí and Tocantins), and the Atlantic Forest (Sergipe). In addition, we tested 80 liver samples of *Pteronotus* spp. from Central America (Panama, Guatemala and El Salvador) and South America (Guyana, Suriname and Venezuela) from the archives of Royal Ontario Museum in Canada (Table 1 and Additional file 1). Blood and tissue samples (BSC/TSC) positive for trypanosomes, and DNA from these samples were preserved in the TCC-USP.

**Table 1:** *Trypanosoma wauwau* and closely related trypanosomes from Neotropical phyllostomid bats and Australian marsupials and rodents included in the phylogenetic tree based on V7V8 SSU rRNA and gGAPDH genes (Fig. 4)

*Trypanosoma*		Host bat^c^		Year	Geographic Origin	
Isolate		Family species			Locality country	
*T. wauwau* cultures (TCC^a^)						
352	ROMO 86	Mor	*Pteronotus parnellii*	2001	Monte Negro/Rondônia	BR
409-413	ROMO 166/156/159/167/163	Mor	*Pteronotus parnellii*	2002	Monte Negro/Rondônia	BR
599/600	HMO 150/152	Mor	*Pteronotus parnellii*	2002	Porto Velho/Rondônia	BR
980-989/1007/	ROMO 01-04/06/08/20/22-24/ 50/56/41/51/48	Mor	*Pteronotus parnellii*	2005	Porto Velho/Rondônia	BR
1008/1019-1023	43/44					
1871/1878	Ptero 6/8	Mor	*Pteronotus gymnonotus*	2009	Porto Velho/Rondônia	BR
1872/1873	Ptero 11/17	Mor	*Pteronotus personatus*	2009	Porto Velho/Rondônia	BR
Archived blood/tissue of *Pteronotus* bats (BSC/TSC^b^)						
PR	100/105	Mor	*Pteronotus gymnonotus*	2006	Itabaiana/Sergipe	BR
VCT	6227/ 6236/ 6238/ 6239/ 6254/ 6379/ 6409	Mor	*Pteronotus gymnonotus*	2009	Parauapebas/Pará	BR
VCT	1103	Mor	*Pteronotus parnellii*	2007	Parauapebas/Pará	BR
VCT	3880	Mor	*Pteronotus parnellii*	2008	Xinguara/Pará	BR
VCT	4330	Mor	*Pteronotus parnellii*	2008	Canaã dos Carajás/Pará	BR
MOL	174	Mor	*Pteronotus parnellii*	2004	Rio Sono/Tocantins	BR
RB	06	Mor	*Pteronotus parnellii*	2010	Ribeirãozinho/Mato Grosso	BR
MN7	07	Mor	*Pteronotus parnellii*	2010	São Vicente/Mato Grosso	BR
ROM	97963/97965	Mor	*Pteronotus parnellii*	1990	Annai/Upper Takutu Upper Essequibo	GY
ROM	102929/102973/102990/103126	Mor	*Pteronotus parnellii*	1994	Surama/Upper Takutu Upper Essequibo	GY
ROM	103420	Mor	*Pteronotus parnellii*	1994	Tropenbos/Upper Demerara-Berbice	GY
ROM	106659	Mor	*Pteronotus parnellii*	1996	Upper Takutu Upper Essequibo	GY
ROM	107348/109024/109292	Mor	*Pteronotus parnellii*	1997	Iwokrama Reserve/Potaro-Siparuni	GY
ROM	111534/111664/111814	Mor	*Pteronotus parnellii*	1999	Iwokrama Forest	GY
					Potaro-Siparuni	
ROM	113739/113823	Mor	*Pteronotus parnellii*	2001	Demerara/Mahaica	GY
ROM	115482/115561	Mor	*Pteronotus parnellii*	2002	Essequibo-West Demerara/Shanklands	GY
ROM	116524/116636/116651	Mor	*Pteronotus parnellii*	2005	Kaieteur National Park Potaro-Siparuni	GY
ROM	99235	Mor	*Pteronotus parnellii*	1991	Petén	GT
ROM	104227	Mor	*Pteronotus parnellii*	1995	Nacional Park Soberania	PA
					Canal Zone	
ROM	104355/104369	Mor	*Pteronotus parnellii*	1995	Parque Nacional Darién	PA
ROM	114151	Mor	*Pteronotus parnellii*	2002	Brownsberg Nature Park/Brokopondo	SR
Trypanosomes of phyllostomid bats: *T.* spp-Neobats						
*T.* sp Neot 1	093AJBohio/134AJCacao/278AJLeon 216AJGuava/300,302AJBCI	Phy	*Artibeus jamaicensis*	2005	-	PA
	RNMO56/63	Phy	*Trachops cirrhosus*	2012	Angicos/Rio Grande do Norte	BR
*T.* sp Neot 2	082AJBohio2/092AJBohio/275AJLeon 173AJGigante/196AJPenaBlanca	Phy	*Artibeus jamaicensis*	2005	-	PA
*T.* sp Neot 3	070AJGuanabano/109AJBohio/240,268,269,282AJLeon/121AJCacao	Phy	*Artibeus jamaicensis*	2005	-	PA
	BACO44/ 46	Phy	*Artibeus lituratus*	2014	Boyacá	CO
Australian trypanosomes			Marsupial and rodent hosts			
*T.* sp	H25		*Macropus giganteus -* kangaroo	1997	-	AU
*T.* sp	G8		*Bettongia penicillata -* woylie	2013	-	AU
*T.* sp	BDA1		*Bettongia lesueur -* woylie	2009	-	AU
*T.* sp	D15/D17/D64		*Trichosurus vulpecula -* possum	2009	-	AU
*T.* sp	BRA2		*Rattus fuscipes -* rodent	2007	-	AU

^a^ TCC, codes of cultures deposited in the Trypanosomatid Culture Collection of the Department of Parasitology, University of São Paulo, Brazil (TCC-USP)

^b^ BSC/TSC, codes of blood and tissue samples deposited in the TCC-USP

^c^ Mor, Mormoopidae, Phy, Phyllostomidae. BR, Brazil; GY, Guyana; GT, Guatemala; PA, Panamá; SR, Suriname; CO, Colombia; AU, Australia

### Barcoding (V7V8 SSU rRNA) of bat trypanosomes in culture and blood samples

The DNA extracted from the cultures of bat trypanosomes using the phenol-chloroform method was used for PCR amplification of the variable V7V8 region of SSU rRNA (~800 bp). To detect the presence of trypanosomes in archived bat samples, we used a nested-PCR that target partial sequence (~561 bp) of the V7V8 SSU rRNA [29]. For the amplification of entire V7V8 SSU rRNA genes, other nested-PCR was developed using the primers 285 F/202R in the first round and the primers 609 F/706R in the second round as reported previously [30].

### Phylogenetic analyses of whole SSU rRNA and gGAPDH genes

The sequences of SSU rRNA and gGAPDH genes were obtained as described previously [30], and alignments were obtained using Clustal X [31] and manually refined. We created the following alignments: a) entire SSU rRNA sequences (~1728 bp) of the novel samples aligned with those from available trypanosomes from bats and other hosts using non-trypanosome trypanosomatids as outgroups [32]; b) concatenated sequences of entire V7V8 SSU rRNA and gGAPDH genes from all trypanosomes of the *T. cruzi* clade using *T. lewisi* as outgroup (Table 1). All the species included in the phylogenetic analyses, and their respective hosts, geographical origins and GenBank accession numbers are provided as Additional files (Table 1 and Additional file 2).

The phylogenies were inferred using the parsimony (P), maximum likelihood (ML) and Bayesian inferences (BI) analyses. The parsimony and bootstrap analyses were carried out using PAUP version 4.0b10 [33] with 500 replicates of random addition sequences followed by branch swapping (RAS-TBR). The ML analyses were performed using RAxML-VI-HPC v.2.2.3 [34] with tree searches performed with GTR model with gamma-distributed rate variation across sites and proportion of invariable sites (GTRGAMMA model) and 500 maximum parsimony-starting trees; the model parameters were estimated in RAxML for the duration of the tree search [32]. Nodal supports were estimated with 500 bootstrap replicates (alignments 1 and 2) in RAxML using GTRGAMMA and maximum parsimony starting trees. The BI analyses were performed in MrBayes v3.1.2 [35] with GTRGAMMA and the first 25 % of the trees from 1 million generations were discarded as burn-in as previously detailed [32].

### Spliced leader (SL) RNA sequences: amplification, sequencing and data analysis

The amplification of the whole SL RNA gene repeats and sequencing of both strands of at least five clones from each isolate, obtained from two independent PCR reactions, were performed as described previously [36]. The alignment of resulting sequences was manually refined. Network genealogy was inferred by SplitsTree v4.11.3 using the neighbour-net method [37]. The analysis of secondary structures was performed as before [4].

### Morphology, growth behaviour and development in mammalian cell cultures, triatomine bugs and mice

We examined blood smears from naturally infected bats and logarithmic and stationary phase cultures obtained with or without the monolayers of Hi-5 insect cells of two selected isolates, one from each genotype (TCC411 and TCC1873). The flagellates smeared in glass-slides were Giemsa-stained. To verify whether the trypanosome differentiated in the supernatant and invaded and developed within mammalian cells, stationary cultures that contained a reasonable number of trypomastigotes were transferred to monolayers of monkey LLC-MK2 cells cultivated at 37 °C, as described previously [4]. The isolates TCC411, TCC413 and TCC599 were assessed for their ability to infect triatomine bugs and Balb/c mice, as described previously [9, 15].

### Transmission (TEM) and scanning (SEM) electron microscopy

For TEM analyses, cultures at mid-log phase from trypanosomes (TCC411 and TCC1873) were fixed with glutaraldehyde, post-fixed in osmium tetroxide, embedded in Spurr’s resin, and examined with a JEOL 100CX electron microscope. For SEM analysis, flagellates fixed with glutaraldehyde were adhered to poly-L-lysine-coated coverslips and processed for observation on a ZEISS DSM 940 microscope as reported before [30].

## Results

### Surveys by haemoculture and isolation in culture of trypanosomes from *Pteronotus* spp

During the surveys of trypanosomes carried out from 2001 to 2009 in the state of Rondonia, 83 *Pteronotus* bats were captured, and the haemoculture (HE) analysis yielded a general prevalence of ~35 %, resulting in 29 cultures of trypanosomes obtained from *P. parnellii* (25), *P. personatus* (2) and *P. gymnonotus* (2) (Table 1). Most of the bats captured in Rondonia were from two shelters, a cave and a river bridge, separated by ~300 km and shared with phyllostomid bats (Fig. 1; Table 1). Cultures of trypanosomes were obtained by HE from bats from different families captured in the two shelters (Additional file 2). *Pteronotus* bats from other Brazilian states and other countries were not examined by haemoculturing.

### The prevalence of trypanosomes in blood/tissues of *Pteronotus* spp. from a wide geographical range

In Brazil, blood/tissue samples of 101 *Pteronotus* bats examined by nested-PCR included samples from *P. parnellii* (56), *P. personatus* (26) and *P. gymnonotus* (19) from the states of Para, Mato Grosso, Maranhão, Goiás, Piauí, Tocantins and Sergipe. We identified only 15 bats positive for trypanosomes (~15 %) probably due to the small size of archived liver samples used for DNA preparation. However, the analysis of 80 archived tissue samples of *P. parnellii* from other countries showed a prevalence of ~32.5 % (26 positive bats). Altogether, we found trypanosomes by nested-PCR in 41of 181 blood/tissues samples: 32 of 136 samples examined from *P. parnellii* and 9 of 19 from *P. gymnonotus.* The details of the host species, geographical origins and trypanosome species, and genotypes detected in the *Pteronotus* bats examined in the present study are shown in Table 1 and in the Additional file 1.

### V7V8 SSU rRNA barcoding revealed a novel trypanosome species in *Pteronotus* bats

**Fig. 2:**
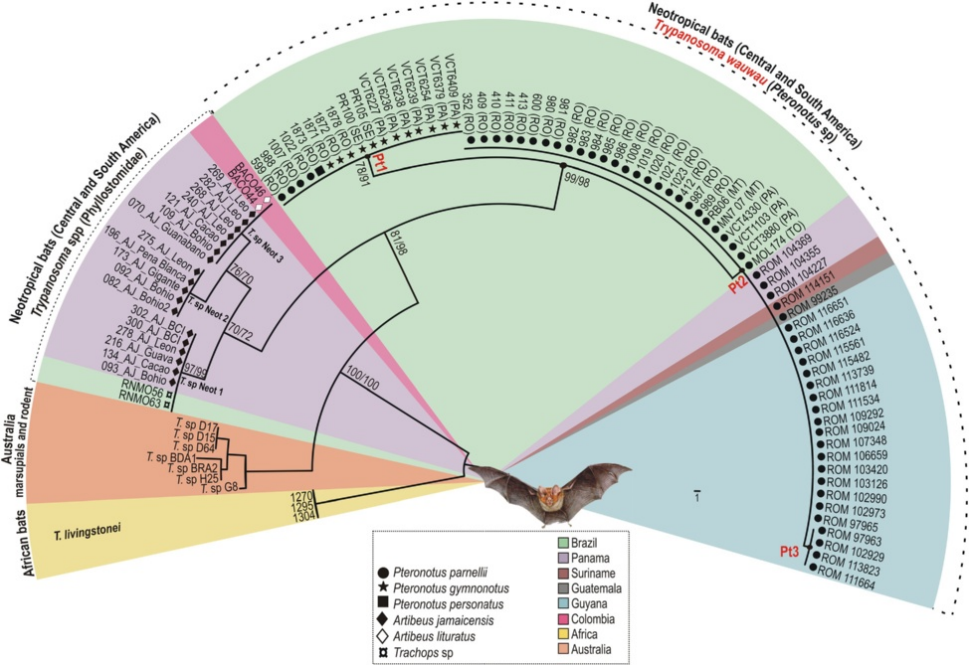
Barcoding and phylogeographical analysis of new trypanosomes from Neotropical bats. Phylogenetic analysis of V7V8 SSU rRNA sequences of trypanosomes from cultures and bat blood/tissue samples from *Pteronotus* and Phyllostomidae bats from Central and South America. The numbers on the nodes are bootstrap values (P/ML) derived from 500 replicates

In the phylogenetic analysis, the V7V8 SSU rRNA sequences from 70 trypanosome samples obtained by HE or from blood/tissue samples of *Pteronotus* spp. formed a strongly supported clade. Despite sharing high similarity (0.4 % of sequence divergence), the sequences were separated into three clusters designated as Pt1-Pt3. The trypanosomes from Brazilian bats clustered in Pt1 and Pt2, whereas Pt3 comprised exclusively the four samples from Guyana (Fig. 2). The small fragment of SSU rRNA (~561 bp) sequenced from the isolates from Guatemala, Suriname, Guyana and Panama lacked the beginning of gene sequences, which contained the sites that distinguished between Pt1 and Pt2, so we were unable to identify the genotypes of these trypanosomes.

The divergences in the barcode sequences separating the trypanosomes of *Pteronotus* bats from the other trypanosomes were as follows: 1) ~5.5 % from the barcode sequences of trypanosomes from Panamanian [11], Colombian and Brazilian phyllostomids (the clade *T.* spp. Neobats), which correspond to several new trypanosome species; 2) ~7.6 % from sequences of the Australian trypanosomes from kangaroo (*T.* sp. H25), possums (*T.* sp. D15, D17 and D64), woylie (*T.* sp. G8 and *T.* sp. BDA1) and bush rats (*T.* sp. BRA2); and 3) ~10 % from *T. livingstonei* of African bats. Therefore, the large genetic distances separating the trypanosomes indicated that the *Pteronotus* isolates are representatives of a new trypanosome species, which was herein named *Trypanosoma wauwau* n. sp.

### Phylogenetic relationships within the clade *T. cruzi* based on whole SSU rRNA and gGAPDH genes

**Fig. 3:**
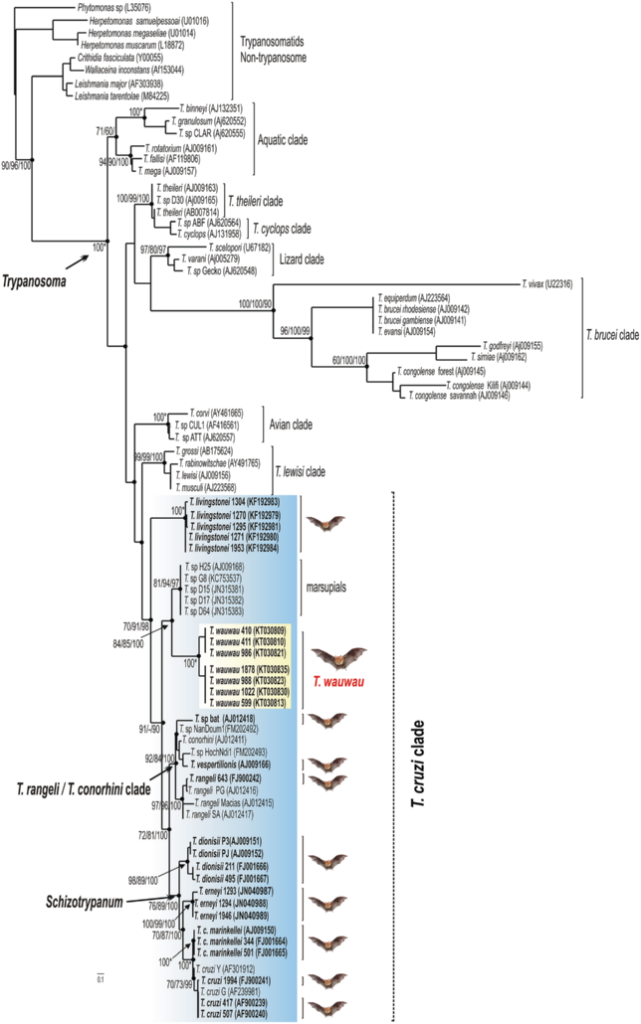
Positioning of *T. wauwau* in the phylogenetic tree of *Trypanosoma*. Phylogenetic tree (ML) inferred using whole SSU rRNA sequences from the trypanosomes of Noctilionoidea Neotropical bats: *T. wauwau,* to date found exclusively in *Pteronotus* bats clustered with the trypanosomes of the clade *T.* spp. Neobats (Panamanian, Colombian and Brazilian Phyllostomidae bats) close to the clade of Australian bats. The analyses included species of all major clades of *Trypanosoma* and trypanosomatids of other genera as outgroups (1.728 characters, –Ln = -8870.359849). The numbers at the nodes correspond respectively to P, ML (500 replicates) and BI support values. Codes within parenthesis are GenBank accession numbers

We selected seven isolates from *Pteronotus* spp. representatives of the genotypes Pt1 and Pt2 for the positioning of *T.wauwau* in the *Trypanosoma* phylogenetic tree using the whole SSU rRNA and gGAPDH sequences. Two isolates from the clade *T.* spp*.* Neobats were also included in the analyses. The phylogenetic trees inferred using these genes exhibited highly congruent topologies, as showed using SSU rRNA sequences alone (Fig. 3), which are the only sequences available in GenBank for all trypanosomes included in the analyses, especially those obtained from blood and tissue samples, whereas most gGAPDH sequences are from cultured trypanosomes.

**Fig. 4:**
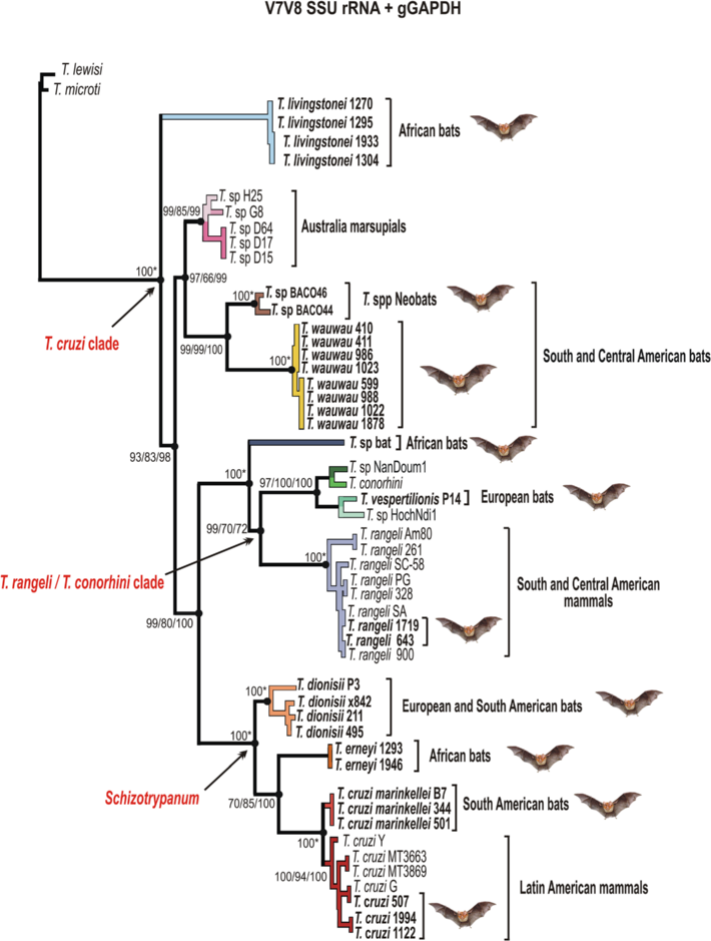
Phylogenetic relationships between *T. wauwau* and the other *T. cruzi* clade trypanosomes. ML phylogenetic analysis based on the concatenated sequences of V7V8 SSU rRNA and gGAPDH genes (1.691 characters, –Ln = 8457.739334) from seven isolates (genotypes Pt1 and Pt2) of *T. wauwau,* two isolates of *T.* spp. Neobats, other 24 bat trypanosomes, and 13 trypanosomes from other mammals. Species of the clade *T. lewisi* were used as outgroup*.* The numbers at the nodes correspond respectively to P, ML (500 replicates) and BI support values

In the better-resolved phylogenetic trees inferred using concatenated SSU rRNA and gGAPDH sequences, the clade of trypanosomes from *Pteronotus* bats was sister to the clade *T.* spp. Neobats, and both formed a clade sister to the Australian clade (Fig. 4). Although the support values for the positioning of these trypanosomes varied depending on the taxa included in the analyses and the methods employed for the inferences, the positioning of *T. wauwau* was consistent in most phylogenetic analyses. In addition, the relationships among other trypanosomes within and outside the *T. cruzi* clade were consistent with our previous phylogenies [3, 4].

The degree of gGAPDH divergences separating between the genotypes Pt1 and Pt2 of the *Pteronotus* trypanosomes were 0.6 %; we are describing a single species with two genotypes to be consistent with other species of the clade such as *T. cruzi*, *T. c. marinkellei, T. dionisii* and *T. rangeli* that comprises an increasing number of divergent genotypes/lineages [2, 3, 5, 15]. The gGAPDH divergences separating *T. wauwau* from related trypanosomes were ~ 9.0 % from the nearest Neotropical trypanosomes of the clade *T.*spp. Neobats, 10 % from the Australian clade, 14.3 and 14.6 % from *T. livingstonei* and *T.* sp.bat, an unnamed and unique species from African megabats, respectively, and 15.5 % from *T. vespertilionis* of European bats. Therefore, the positioning into the phylogenetic trees, and the highly relevant degree of sequence divergence from other trypanosomes strongly supported the description of *Trypanosoma wauwau* n. sp.

### *Trypanosoma wauwau* n. sp. was tightly linked to *Pteronotus* bats

The analysis of 264 bats of the genus *Pteronotus,* including 83 cultures and 181 blood/tissue samples, revealed a high prevalence of infection with *T. wauwau* (average of ~26,5 %). This trypanosome species was not detected in a large sampling of bats from other genera and families investigated so far by our and other research groups in this and in previous studies [9, 11, 15–19]. Phyllostomid bats captured in shelters shared with *Pteronotus* in Rondônia, western Amazonia, were identified as *T. c. marinkellei* and *T. dionisii* [9]. Previous studies suggested some degree of specificity of trypanosome species to certain bat taxa. For instance, *T. c. marinkellei* appear to be composed of divergent trypanosomes and genotypes related to different genera of the Phyllostomidae family [9]. Also suggesting some host-specificity, *T. livingstonei* was identified in African bats of the closely related genera *Rhinolophus* and *Hipposideros*, whereas sympatric bats of Molossidae harboured *T. erneyi* [3, 4]. However, host-specificity of bat trypanosomes were still limited to data from a few surveys in general focused in the more abundant and easier to capture bat species, then precluding any strong associations of trypanosome species with bat hosts and geography.

Notably, other than *T. wauwau, Pteronotus* bats are apparently infected by very few other trypanosome species, despite the presence of *T. cruzi* in species of *Pteronotus* in Brazil [5, 38] and Mexico [39]. In contrast, species of diverse genera of Phyllostomidae that shared areas and shelters with *Pteronotus* were infected with a wide range of trypanosomes, including *T. dionisii*, *T. c. marinkellei* and unnamed species of the clade *T.* spp. Neobats, as shown in this (Additional file 2) and previous studies [9, 11].

### High conservation of transcripts and structures of SL RNAgene repeat of *T. wauwau* and trypanosomes from Australia and Africa

The SL RNA genes have been used as taxonomic markers for trypanosomatids because the repeats of SL RNA vary in both length and sequence, and the different species exhibited highly conserved exons, moderately conserved introns and highly variable intergenic sequences. Shared by all SL RNA structures, the Y-shaped topology is formed by three stem-loops and a bifurcation point variable according to the species/genotypes [4, 15, 36, 40].

**Fig. 5:**
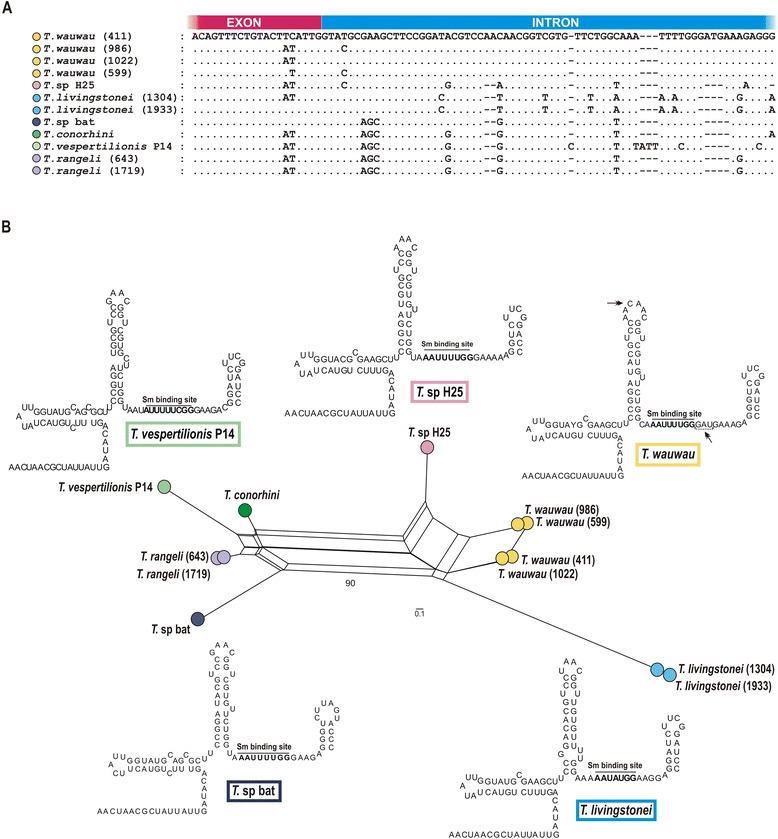
The SL RNA primary and secondary structures of *T. wauwau* and its closest trypanosomes*.* **a**, Aligned sequences of the SL RNA transcripts from *T. wauwau* (Pt1 and Pt2 genotypes), and other *T. cruzi* clade trypanosomes. **b**, Network of SL RNA transcript sequences and almost identical secondary structures shared by Neotropical *T. wauwau* and Australian *T.* sp H25 (differences are indicated by *arrows* in the *T. wauwau* SL structure), and highly similar to that of *T. livingstonei* from African bats. Numbers in nodes correspond to bootstrap values estimated by 500 replicates using the same parameters optimized for network inferences

We determined the primary sequences of cloned full-length SLRNA repeats of four isolates of *T. wauwau*. The SL RNA repeats varied in length, ~722 bp and ~702 bp for the *T. wauwau* genotypes Pt1 and Pt2, respectively, in addition to SNPs, microsatellites and insertions/deletions in the intergenic regions that distinguished the two genotypes. The intergenic regions of *T. wauwau* could not be aligned with confidence with those from any other trypanosome species (data not shown). Notable, *T. wauwau* shared highly conserved transcript sequences, and almost identical secondary structures when compared with those from its closest relatives *T.* sp H25 (SL RNA characterized in the present study) and *T. livingstonei* (Fig. 5a, b) [4].

### Behaviour of *T. wauwau* inoculated in mice and triatomine bugs

Similar to the behaviour shown previously for *T.* sp*.* H25 [29] and *T. livingstonei* [4], *T. wauwau* did not develop within mammalian (human and monkey) cells in vitro, and was unable to infect mice as determined by negative HE and PCR tests of mice blood samples done from 2 to 30 days after the inoculation of cultured trypomastigotes of *T. wauwau*.

*T. wauwau* was not infective to triatomines (*Rhodnius robustus, Rhodnius neglectus* and *Triatoma infestans*), which destroyed the parasites in their gut and haemolymph. Similar results were obtained for *T. dionisii*, *T. erneyi* and *T. livingstonei* [3, 4, 9]. The high prevalence of bats infected with *T. wauwau* suggested that this species should be transmitted by common vectors and routes. The *Pteronotus* bats captured were in general heavily infested with ectoparasites such as hippoboscid flies and ticks, but cimicids were not found in these bats. Cave-dwelling sand flies can be the vectors of bat trypanosomes as previously indicated for *T. leonidasdeanei* in Central America [41], and suggested by prevalent trypanosome infection in sand flies usually associated with bats [42]. Studies of ectoparasites and sand flies associated with bats are required, as done to demonstrate that cimicids cyclically transmit *T. dionisii* and *T. vespertilionis* in Africa and Europe [43]. However, mechanical transmission through the bites of ectoparasites, and oral infection through the ingestion of ectoparasites, are very probable among bats that live in colonies, and share grooming and feeding on the ectoparasites.

### Morphology of blood and culture developmental forms and growth behaviour of *T. wauwau*

**Fig. 6:**
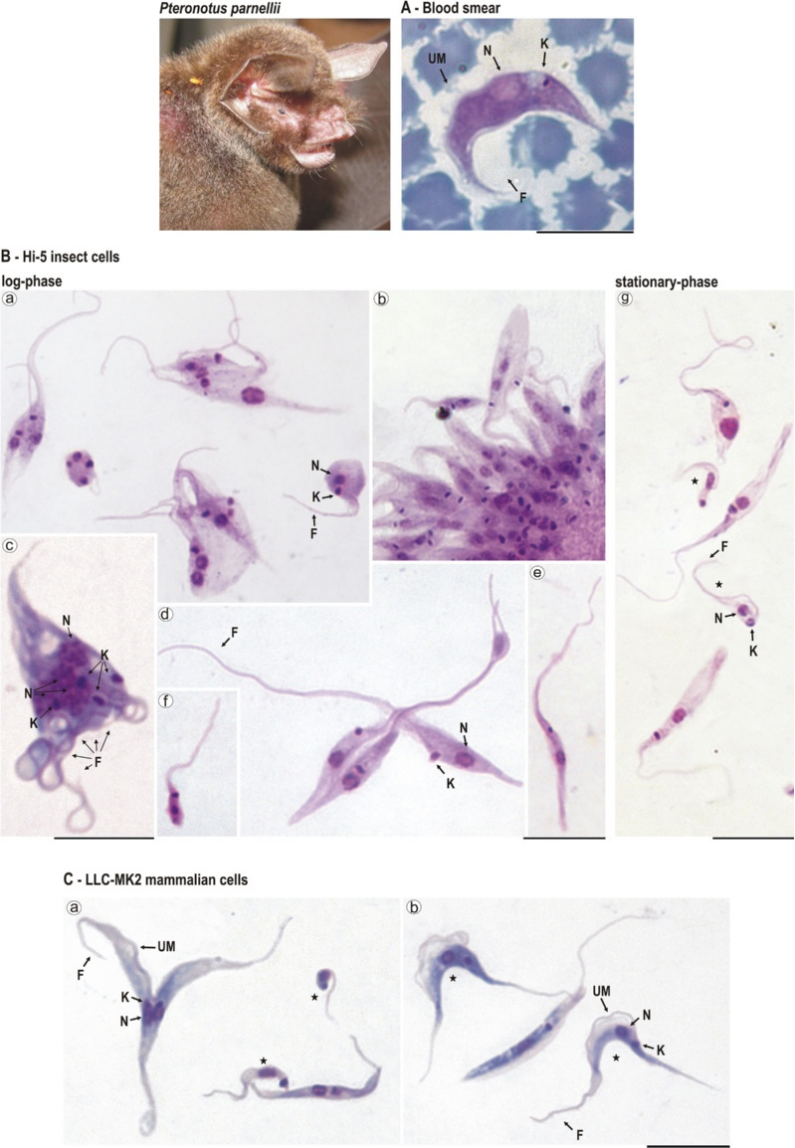
*Pteronotus parnellii* and developmental forms of *T. wauwau*: Light microscopy of Giemsa-stained forms: **A**, trypomastigotes in bat blood smear. **B**, flagellates co-cultivated with Hi-5 insect cells: Supernatants of early cultures showing small and rounded division forms (a, b, c), multiple fission forms united by the posterior extremity exhibiting various nuclei, kinetoplasts and flagella (a, c), rosettes of epimastigotes attached by the flagella (b), epimastigotes largely varying in shape and size (b, d-f), log-phase regular epimastigote that multiply by binary fission (d), small trypomastigotes with terminal kinetoplast of stationary cultures (g). **C**, Epimastigotes (a, b) and trypomastigotes (b) with noticeable undulant membrane, and small trypomastigotes (a) in the supernatant of LLC-MK2 mammalian cells at 37 °C. Trypomastigotes are indicated by *black stars*. Nucleus (N); Kinetoplast (K); Flagellum (F), Undulant Membrane (UM). Scale bars: 10 μm

The parasitemia was very low in all bats examined, even in blood samples of bats that generated positive haemocultures. The blood smears stained with Giemsa of *Pteronotus* bats showed scarce large trypomastigote forms with a wide and striated body, pointed posterior end, a noticeable undulating membrane, and a short free flagellum. The small kinetoplast occupied a lateral position adjacent to the rounded and nearly to the central nucleus (Fig. 6a). The trypomastigotes in the *Pteronotus* blood smears resembled those of *T. leonidasdeanei* and *T. pessoai* in Central and South American bats [41, 44], and the trypomastigotes found in blood smears of African bats infected with *T. heybergi* and *T. livingstonei* [4, 45]. Interestingly, blood trypomastigotes of *T. wauwau* were also quite similar to those of *T.* sp. from the Australian marsupial *Trichosurus vulpecula*, which clustered together with *T.* sp. H25 in the clade *T. cruzi*; so far the blood forms of *T.* sp. H25 remain undescribed [29, 46].

The developmental and morphological analyses of *T. wauwau* co-cultivated with Hi-5 cells showed initially spheromastigotes that multiply by binary or multiple and irregular fissions (Fig. 6Ba) generating rosettes of epimastigotes attached by their flagella (Fig. 6Bb), and large forms exhibiting various flagella (Fig. 6Bc). The free epimastigotes varied largely in shape and size (Fig. 6Bb, d–f), and the more common log-phase forms (Fig. 6Bd) ranged in length from 11.0 to 35.7 μm (average of 23.8 μm) and in width from 1.0 to 5.2 μm (average of 2.3 μm). These forms exhibited a punctual kinetoplast, in general, not adjacent to the central nucleus, and a long flagellum (average 13.0 μm), but undulant membrane was unnoticeable (Fig. 6Bd,e). The stationary cultures exhibited small trypomastigotes with a rounded posterior extremity and terminal kinetoplast (Fig. 6Bg).

The co-cultivation of *T. wauwau* with a monolayer of LLC-MK2 at 37 °C displayed, in the supernatants of the cultures, epimastigotes (Fig. 6Ca) that differentiate to trypomastigotes (Fig. 6Ca,b) of the two main morphotypes: 1) long and wide multiplicative forms with pointed posterior end, noticeable undulant membrane, and punctual kinetoplast, and 2) small trypomastigotes with a large terminal kinetoplast (Fig. 6Ca,b). Although some rounded flagellates resembling amastigote forms were detected in the beginning of the cultures inside of a few cells, they were not able to multiply.

### Morphological and ultrastructural features of *T. wauwau* assessed by electron microscopy

**Fig. 7:**
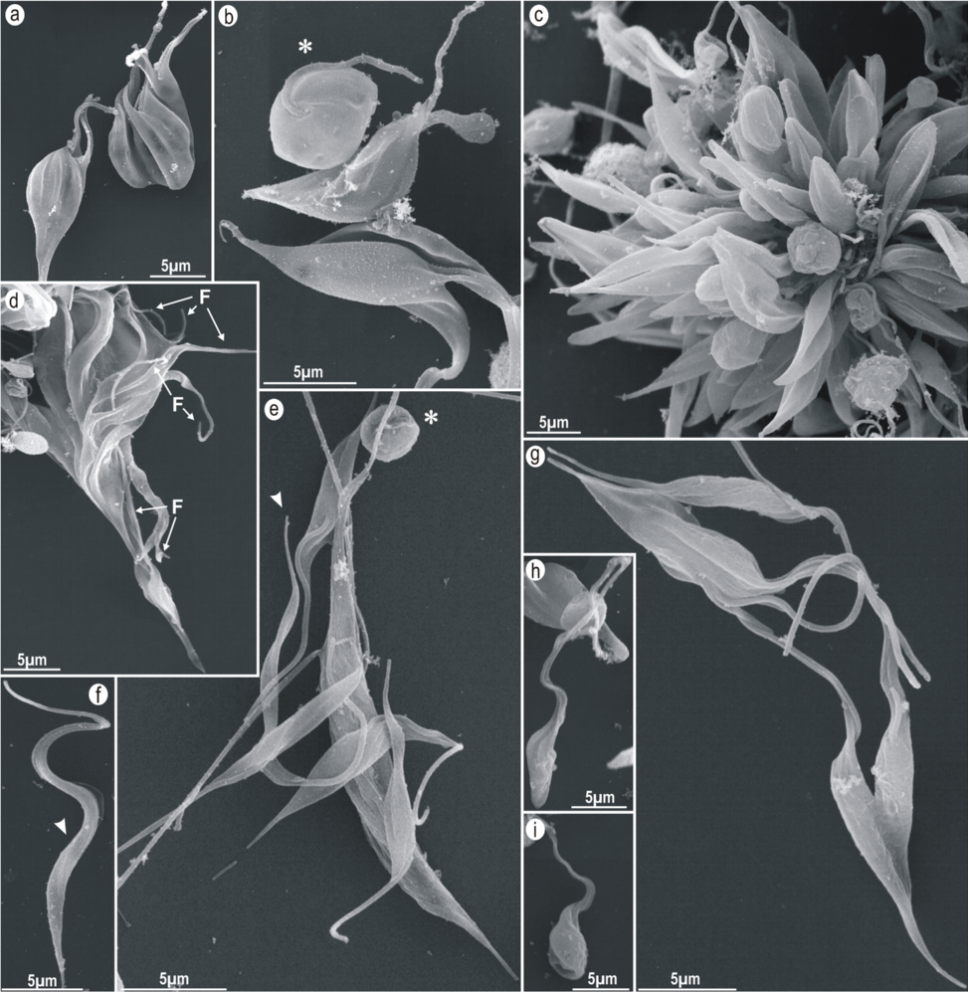
Morphology of *T. wauwau* developmental forms in culture assessed by SEM. Small dividing forms and rounded flagellates common in early cultures (**a**, **b**),rosettes of epimastigotes attached by the flagella (**c**), large multiple fission form showing the anterior ends and the flagella of many parasites that remained united by the posterior extremity (**d**), epimastigotes of variable size and shape likely originated from multiple and irregular division forms (**e**, **f**, **h**), log-phase regular epimastigotes and binary division (**g**), small flagellate resembling the trypomastigotes of stationary-phase cultures (**i**)

**Fig. 8:**
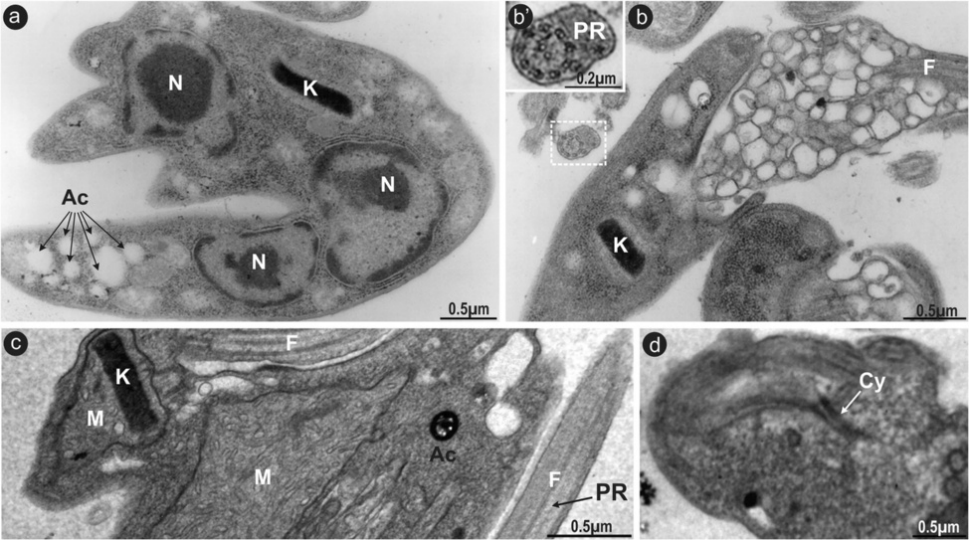
Ultrastructural features of *T. wauwau* revealed by TEM microscopy. Cultured epimastigotes: transversal section showing three nuclei with large and condensed nucleolus and a single kinetoplast (**a**); acidocalcisomes (**b**), flagellum with a conspicuous paraxial structure (**b’**, **c**), highly compacted disk-shaped kinetoplast (**b**, **c**), enlarged mitochondria filled with many cristae (**c**), structure resembling a short cytostome-cytopharynx complex (**d**). Nucleus (N), Kinetoplast (K), Flagellum (F), Acidocalcisomes (Ac), Mitochondria (M), Cytostome (Cy), Paraxial structure (PR)

The analyses of *T. wauwau* by SEM showed small rounded forms that divided by multiple irregular fissions (Fig. 7a,b) forming rosettes of epimastigotes united by the flagella (Fig. 7c) or large forms likely resulting from multiple and incomplete fissions (Fig. 7b,d). The cultures also exhibited free epimastigotes (Fig. 7e–h), which multiplies by binary fission and differentiate to epimastigotes pointed at posterior end, lacking visible undulant membrane, and exhibiting a long flagella (Fig. 7g). Few small trypomastigotes were also observed (Fig. 7i). The TEM ultrastructural analysis revealed a set of morphological features unique of *T. wauwau*: unusual large, rounded and condensed nucleolus in dividing epimastigotes often showing more than two nuclei that divided before the kinetoplast (Fig. 8a), the flagellum exhibiting a conspicuous paraxial structure (Fig. 8b,c), flagellar pockets showing many vesicles (Fig. 8b), large numbers of acidocalcisomes (Fig. 8a), enlarged mitochondria with many cristae (Fig. 8c), kDNA fibrils arranged in a highly compacted disk-shaped kinetoplast (Fig. 8b,c), and an electron-dense structure that resembled a short cytostome-cytopharynx complex (Fig. 8d). The ultrastructural features displayed by *T. wauwau* were more similar to those exhibited by *T. livingstonei* [4] than to the general features shared by all bat trypanosomes of the subgenus *Schizotrypanum* as revealed in previous studies for *T. cruzi*, *T. dionisii* and *T. erneyi* [3, 4].

### Taxonomic summary

Phylum Euglenozoa Cavalier-Smith, 1981; Class Kinetoplastea Honigberg, 1963; Order Trypanosomatida Hollande, 1952; Family Trypanosomatidae Doflein, 1951; Genus *Trypanosoma* Gruby, 1843.

#### New species description

*Trypanosoma wauwau* n. sp.

##### Type material

Hapantotype: the culture of the isolate TCC411 cryo preserved at TCC-USP. Paratypes: the cultures of the isolates TCC599, 988, 1007, 1022, 1871-1873 and 1878, all identified as genotype Pt1 of *T. wauwau*. The cultures of the isolates TCC352, 409-413, 600, 980-987, 989, 1008, 1019-1021 and 1023 are considered the genotype Pt2 of *T. wauwau*.

##### Type host

Chiroptera, Mormoopidae, *Pteronotus parnellii*.

##### Additional host

Chiroptera, Mormoopidae, *Pteronotus gymnonotus* and *Pteronotus personatus.*

##### Locality

Brazil, state of Rondonia, Amazonia.

##### Additional localities in Brazil

States of Pará, Mato Grosso, Tocantins and Sergipe.

##### Additional countries

Guyana, Suriname, Panama and Guatemala.

##### Morphology

The blood trypomastigotes are large and wide with body striations, small kinetoplast and frilled undulating membrane. The epimastigotes predominating in log-phase cultures are long and pointed at posterior ends (averaging 23.8 μm in length and 2.3 in width), in general, the kinetoplast is laterally positioned and not adjacent to the nucleus, and the flagellum is long (average 13.0 μm). All forms are shown in the Figs. 6 and 7.

##### Species diagnosis

DNA sequences (isolate TCC411) unique to *T. wauwau* deposited in GenBank (accession numbers): SSU rRNA (KT030810), gGAPDH (KT030800) and SL gene (KT368810).

##### Etymology

The name *Trypanosoma wauwau* n. sp. was adopted because this species was firstly discovered in bats captured in the Brazilian state of Rondonia, Western Amazonia, near the land-dwelling of the endangered Brazilian indigenous people Uru-Eu-Wau-Wau.

##### Species depository

The cultures of *T. wauwau* are all cryopreserved at the Trypanosomatid Culture Collection of the University of São Paulo, TCC-USP. Giemsa-stained smears of cultures and blood samples of bats infected with *T. wauwau*, and DNA from cultures and *T. wauwau*-infected bat blood/tissue samples are also conserved at TCC-USP. *Trypanosoma wauwau* n. sp. was registered in ZooBank, the online registration system for the ICZN, under the code: to urn:lsid:zoobank.org:pub: 67EBC3EB-35B4-4645-B45A-F12CA818DC09.

## Discussion

In this study, we described the prevalent *Trypanosoma wauwau* n. sp. that infected Neotropical bats of the genus *Pteronotus* (Mormoopidae) and nested into the *T. cruzi* clade, then supporting the bat-seeding hypothesis proposed for the origin of this clade [1, 3, 4]. Comprehensive surveys of bat trypanosomes strongly linked *T. wauwau* to *Pteronotus* bats. The phylogeographical analysis of the *T. wauwau* isolates from wide geographical range revealed two main genotypes infecting three species of *Pteronotus, P. parnellii*, *P. personatus* and *P. gymnonotus,* across Central and South America. Bats of *Mormoops,* the other genus of the Mormoopidae, were not examined to determine whether *T. wauwau* can parasitize bats of the entire family.

In the SSU rRNA and gGAPDH phylogenies, *T. wauwau* was sister to the clade composed of trypanosomes from Panama [11], Brazil and Colombia, all from Neotropical Phyllostomidae bats and clustered in the clade *T.* spp. Neobats. The positioning of *T. wauwau* and *T.* spp. Neobats as the most basal trypanosomes of Neotropical bats, and closer to Australian than to other Neotropical trypanosomes is very relevant to the evolutionary history of the *T. cruzi* clade*.* Corroborating previous studies, *T. livingstonei* from African bats remained at the edge of this clade [4]. However, the eventual positioning of New World trypanosomes in more basal positions can change the hypothesis of Old World origin for the *T. cruzi* clade.

Previously to the bat-seeding hypothesis, a southern super-continent hypothesis was suggested by the relationships and host distribution of the *T. cruzi* clade trypanosomes, especially by the positioning of the Australian *T.* sp. H25 (kangaroo) at the edge of the clade. According to this scenario, *T. cruzi* and related parasites could be primarily evolved in marsupials of South America, Antarctica and Australia [47]. Contradicting the southern super-continent hypothesis, African trypanosomes of civets (carnivorous) and monkeys nested into the *T. cruzi* clade, showing that the species of this clade were also present in African terrestrial mammals, in addition to bats [10].

Currently proposed scenarios suggested multiple movements of marsupials between Australia and South America, which remained connected by Antarctica until ~35 mya. There is also evidence that bats and a few rodents were the only placental mammals that successfully colonized Australia after its complete isolation, and before the animals brought by humans [48, 49]. The Australian trypanosomes within the *T. cruzi* clade were from kangaroo, woylie and possum, marsupials of the order Diprodontia of the superorder Australidelphia [1, 2, 29, 50–53]. The order Microbiotheria, which contains a single extant species, is the only Neotropical representative of Australidelphia. New World marsupials (Ameridelphia) are common hosts of *T. cruzi, T. rangeli* and other trypanosome species [45]. Noteworthy, intra erythrocytic parasites of Sarcocystidae molecularly identified from the South American and Australian marsupials shared a common ancestor [54]. However, phylogenetic studies revealed that trypanosomes from Australian marsupials are unrelated to one another, some species showed to be more related to trypanosomes of other hosts outside Australia, and so far no species could be linked to South American marsupials [50, 52, 53].

Despite old reports of trypanosomes infecting Australian bats, including *T. pteropi* showing blood trypomastigotes resembling those of the *Schizotrypanum* species [45, 53, 55], only recently trypanosomes from Australian bats began to be molecularly characterized, and *T. vegrandis,* a species previously reported in a range of non-volante mammals (woylie, kangaroo, bandicoot and wallaby) was identified in bats (*Pteropus scapulatus*, *Nyctophilus geoffroyi* and *Chalinolobus gouldii*). *T. vegrandis*, however, is apparently restricted to Australia, and was not nested into the *T. cruzi* clade [53, 56].

*T. wauwau* and the clade *T.* spp. Neobats, an assemblage of several unnamed trypanosome species, were found, respectively, in the Neotropical Mormoopidae and Phyllostomidae families of Noctilionoidea, a superfamiliy with basal groups limited to two extant species of each non-Neotropical Myzopodidae and Mystacinidae families that once flourished in Australia and Africa, respectively [23–27]. Likely, Noctilionoidea may have had their origin in eastern Gondwana, and then dispersed from Africa into Australia from where they could have migrated across Antarctica to South America to give origin to Neotropical noctilionoids [27]. It is tempting to speculate that the ancestors of Noctilionoidea bats carrying trypanosomes of the *T. cruzi* clade once inhabited Australia, and may have been introduced into South America.

## Conclusions

Here, we described *Trypanosoma wauwau* n. sp. of Neotropical *Pteronotus* bats and nested into the *T. cruzi* clade supporting the bat-seeding hypothesis. The findings from the present study suggest a link of Australian trypanosomes with newly discovered Neotropical bat trypanosomes support an evolutionary scenario whereby a lineage of the *T. cruzi* clade may have expanded into Australian mammals. Accordingly, trypanosomes from indigenous Australian mammals within the clade *T. cruzi* likely evolved from a bat trypanosome. Strongly supporting this hypothesis, a new trypanosome species found in an Australian bat (*Pteropus scapulatus*) showed to be related to *T. rangeli* [56, 57]. Therefore, besides the ancient great radiation of bats throughout the Word and more recent movements of bats across the land bridge of the Bering Strait and quite large oceanic barriers [1, 2, 4], a route in the southern supercontinent may also have played an important role in the dispersion of bats carrying *T. cruzi* clade trypanosomes. Our findings contribute to the discussion on the two competing biogeographical hypotheses: whether the ancestor trypanosomes of the clade *T. cruzi* originated in the New World or Old Word bats. The results gathered to date are more consistent with an Old World origin of the bat trypanosome ancestor of the *T. cruzi* clade. The present study provides relevant insights into the origin, dispersion, host-colonization and speciation of trypanosomes that shaped the *T. cruzi* clade*.* However, improved knowledge about Australian, African, and Neotropical trypanosome bats, as well as comprehensive molecular studies of bat trypanosomes from the Nearctic and Palearctic can be valuable to understand the origin and global distribution of *T. cruzi* clade trypanosomes, and to shed more light on the evolution of these intriguing parasites and the emergence of human pathogens.

## Additional files

Additional file 1: Table S1. Prevalence of *Trypanosoma wauwau* and geographical origin of *Pteronotus* spp. examined in this study. (DOC 114 kb) Additional file 2: Table S2. The isolates of *Trypanosoma wauwau* and trypanosome species of the *T. cruzi* clade included in this study: host species, geographic origin and GenBank accession numbers of gene sequences employed in the phylogenetic inferences. (DOC 254 kb)
