# Supplementary material for: New insights into the evolution of the Trypanosoma cruzi clade provided by a new trypanosome species tightly linked to Neotropical Pteronotus bats and related to an Australian lineage of trypanosomes
Source: Parasit Vectors. 2015 Dec 23;8:657. doi: 10.1186/s13071-015-1255-x (PMC4690318; doi:10.1186/s13071-015-1255-x)
Supplement: Additional file 1: Table S1. — Prevalence of Trypanosoma wauwau and geographical origin of Pteronotus spp. examined in this study. (DOC 114 kb) [file 13071_2015_1255_MOESM1_ESM.doc]

**Additional File 1**

**Table S1. Prevalence of *Trypanosoma wauwau* and geographical origin of *Pteronotus* spp.**

| **Geographical origin** | |  | **Biome** | ***Pteronotus* spp.**  **No. of individuals: examined/positive^a^** | | |  |
| --- | --- | --- | --- | --- | --- | --- | --- |
| **Locality** | **State** | **Country** |  | ***P. parnellii*** | ***P. personatus*** | ***P. gymnonotus*** | **Total** |
|  |  |  |  |  |  |  |  |
| Monte Negro | Rondônia | BR | AM | 21/6 | - | *-* | 21/6 |
| Porto Velho | Rondônia | BR | AM | 42/19 | 16/2 | 4/2 | 62/23 |
| Carajás, Parauapebas, Xinguara | Pará | BR | AM | 26/3 | 18/0 | 12/7 | 56/10 |
| Aripuanã | Mato Grosso | BR | AM | 4/0 | *-* | *-* | 4/0 |
| Paranaíta | Mato Grosso | BR | AM | 2/0 | *-* | *-* | 2/0 |
| Serra das Araras | Mato Grosso | BR | CE | 4/0 | *-* | 2/0 | 6/0 |
| Amarante do Maranhão | Maranhão | BR | AM/CE | 1/0 | *-* | *-* | 1/0 |
| Minaçu | Goiás | BR | CE | 1/0 | - | - | 1/0 |
| Ribeiraozinho | Mato Grosso | BR | CE | 2/1 | - | - | 2/1 |
| Rondonópolis | Mato Grosso | BR | CE | 1/0 | - | - | 1/0 |
| Jangada | Mato Grosso | BR | CE | 4/0 | - | 1/0 | 5/0 |
| Uruçuí | Piauí | BR | CE | 1/0 | - | - | 1/0 |
| Parnarama | Maranhão | BR | AM/CE | 1/0 | - | - | 1/0 |
| Rio Sono | Tocantins | BR | CE | 1/1 | - | - | 1/1 |
| Conceição do Tocantins | Tocantins | BR | CE | 1/0 | *-* | *-* | 1/0 |
| Cuiabá | Mato Grosso | BR | CE | 1/0 | - | - | 1/0 |
| São Vicente | Mato Grosso | BR | CE | 6/1 | - | - | 6/1 |
| Itabaiana | Sergipe | BR | AF | - | 8/0 | 4/2 | 12/2 |
| Kuwaima Fall | Cuyuni-Mazaruni | GY | - | 1/0 | - | - | 1/0 |
| Namai Creek | Cuyuni-Mazaruni | GY | - | 3/0 | - | - | 3/0 |
| Ceiba Biological Center | Demerara – Mahaica | GY | - | 6/2 | - | - | 6/2 |
| Madewini Creek Bridge | Demerara – Mahaica | GY | - | 1/0 | - | - | 1/0 |
| Mango Landing, Corentyne River | East Berbice-Corentyne | GY | - | 2/0 | - | - | 2/0 |
| Shanklands | Essequibo Islands-West Demerara | GY | - | 4/2 | - | - | 4/2 |
| Cow Fly Camp, Iwokrama Reserve | Potaro-Siparuni | GY | - | 1/0 | - | - | 1/0 |
| Iwokrama Reserve, Burro Burro River, Kurupukari | Potaro-Siparuni | GY | - | 1/1 | - | - | 1/1 |
| Iwokrama Reserve, Kurupukari | Potaro-Siparuni | GY | - | 2/0 | - | - | 2/0 |
| Iwokrama Reserve, Siparuni River, Kurupukari | Potaro-Siparuni | GY | - | 1/0 | - | - | 1/0 |
| Kabukalli Landing, Iwokrama Forest | Potaro-Siparuni | GY | - | 3/2 | - | - | 3/2 |
| Kaieteur National Park, Kaieteur Falls | Potaro-Siparuni | GY | - | 6/2 | - | - | 6/2 |
| Kaieteur National Park, Menzies Landing | Potaro-Siparuni | GY | - | 3/1 | - | - | 3/1 |
| Mount Ayanganna, Toe Slope Camp | Potaro-Siparuni | GY | - | 1/0 | - | - | 1/0 |
| Mount Daniel Cutline, Iwokrama Forest | Potaro-Siparuni | GY | - | 2/0 | - | - | 2/0 |
| Pakatau Falls, Siparuni River, Iwokrama Reserve | Potaro-Siparuni | GY | - | 1/1 | - | - | 1/1 |
| Sand Stone, Iwokrama Forest | Potaro-Siparuni | GY | - | 1/1 | - | - | 1/1 |
| Iwokrama Reserve, Turtle Mountain, Kurupukari, | Potaro-Siparuni | GY |  | 1/1 | - | - | 1/1 |
| Tropenbos, Mabura Hill | Upper Demerara-Berbice | GY | - | 2/1 | - | - | 2/1 |
| West Pibiri, Mabura | Upper Demerara-Berbice | GY | - | 5/0 | - | - | 5/0 |
| Surama | Upper Takutu-Upper Essequibo | GY | - | 7/4 | - | - | 7/4 |
| Annai | Upper Takutu-Upper Essequibo | GY | - | 2/2 | - | - | 2/2 |
| Kamoa River, Gunn's Strip | Upper Takutu-Upper Essequibo | GY | - | 2/1 | - | - | 2/1 |
| Karanambo | Upper Takutu-Upper Essequibo | GY | - | 1/0 | - | - | 1/0 |
| Quarter Mile Landing, Rupununi River, Annai | Upper Takutu-Upper Essequibo | GY | - | 1/0 | - | - | 1/0 |
| Poptun | El Petén | GT | - | 1/1 | - | - | 1/1 |
| Tikal | El Petén | GT | - | 2/0 | - | - | 2/0 |
| Campo los Guacamayos | El Petén | GT | - | 2/0 | - | - | 2/0 |
| El Remate | El Petén | GT | - | 1/0 | - | - | 1/0 |
| Río Uyus | El Progreso | GT | - | 1/0 | - | - | 1/0 |
| Parque Nacional Darién | Darien | PA | - | 2/2 | - | - | 2/2 |
| Parque Nacional Soberania | Canal Zone | PA | - | 1/1 | - | - | 1/1 |
| Brownsberg Nature Park | Brokopondo | SR | - | 1/1 | - | - | 1/1 |
| Brownsberg Nature Park | Brokopondo | SR | - | 2/0 | - | - | 2/0 |
| Bakhuis | Sipaliwini | SR | - | 2/0 | - | - | 2/0 |
| Parque Nacional Montecristo, Bosque Nebuloso | Santa Ana | SV | - | 3/0 | - | - | 3/0 |
| Pozon, 50 km NE of Puerto Ayacucho | Amazonas | VE | - | 2/0 | - | - | 2/0 |
|  |  |  | **Total** | **199/57** | **42/2** | **23/11** | **264/70** |

BR, Brazil; GY, Guyana; GT, Guatemala; PA; Panamá; SR, Suriname; SV, El Salvador; VE, Venezuela. Brazilian biomes: AM, Amazônia; CE, Cerrado; AF, Atlantic Forest; ^a^ Detection of trypanosomes by haemoculture of bats from Rondônia and by Nested-PCR in samples from other places.
