# Supplementary material for: New insights into the evolution of the Trypanosoma cruzi clade provided by a new trypanosome species tightly linked to Neotropical Pteronotus bats and related to an Australian lineage of trypanosomes
Source: Parasit Vectors. 2015 Dec 23;8:657. doi: 10.1186/s13071-015-1255-x (PMC4690318; doi:10.1186/s13071-015-1255-x)
Supplement: Additional file 2: Table S2. — The isolates of Trypanosoma wauwau and trypanosome species of the T. cruzi clade included in this study: host species, geographic origin and GenBank accession numbers of gene sequences employed in the phylogenetic inferences. (DOC 254 kb) [file 13071_2015_1255_MOESM2_ESM.doc]

**Additional file 2**

**Table S2: The isolates of *Trypanosoma wauwau* and trypanosome species of the *T. cruzi* clade included in this study: host species, geographic origin and GenBank accession numbers of gene sequences.**

| ***Trypanosoma* sp**  **TCC^a^** | |  | **Host Origin** | **Year** | **Geographic Origin** |  | **GenBank Acession number^b^** | | |
| --- | --- | --- | --- | --- | --- | --- | --- | --- | --- |
| ***T. wauwau* isolates** | |  |  |  |  |  | **SSU rRNA** | **GAPDH** | **SL** |
| 352 | ROMO 86 | bat | *Pteronotus parnellii* | 2001 | Monte Negro / Rondonia | BR | **KT030807** | **-** | - |
| 409 | ROMO 166 | bat | *Pteronotus parnellii* | 2002 | Monte Negro / Rondonia | BR | **KT030808** | - | - |
| 410 | ROMO 156 | bat | *Pteronotus parnellii* | 2002 | Monte Negro / Rondonia | BR | **KT030809** | **KT030799** | - |
| 411 | ROMO 159 | bat | *Pteronotus parnellii* | 2002 | Monte Negro / Rondonia | BR | **KT030810** | **KT030800** | **KT368810** |
| 412 | ROMO 167 | bat | *Pteronotus parnellii* | 2002 | Monte Negro / Rondonia | BR | **KT030811** | - | - |
| 413 | ROMO 163 | bat | *Pteronotus parnellii* | 2002 | Monte Negro / Rondonia | BR | **KT030812** | - | - |
| 599 | HMO 150 | bat | *Pteronotus parnellii* | 2002 | Porto Velho / Rondonia | BR | **KT030813** | **KT030803** | **KT368813** |
| 600 | HMO 152 | bat | *Pteronotus parnellii* | 2002 | Porto Velho / Rondonia | BR | **KT030814** | - | - |
| 980 | ROMO 01 | bat | *Pteronotus parnellii* | 2005 | Porto Velho / Rondonia | BR | **KT030815** | - | - |
| 981 | ROMO 02 | bat | *Pteronotus parnellii* | 2005 | Porto Velho / Rondonia | BR | **KT030816** | - | - |
| 982 | ROMO 03 | bat | *Pteronotus parnellii* | 2005 | Porto Velho / Rondonia | BR | **KT030817** | - | - |
| 983 | ROMO 04 | bat | *Pteronotus parnellii* | 2005 | Porto Velho / Rondonia | BR | **KT030818** | - | - |
| 984 | ROMO 06 | bat | *Pteronotus parnellii* | 2005 | Porto Velho / Rondonia | BR | **KT030819** | - | - |
| 985 | ROMO 08 | bat | *Pteronotus parnellii* | 2005 | Porto Velho / Rondonia | BR | **KT030820** | - | - |
| 986 | ROMO 20 | bat | *Pteronotus parnellii* | 2005 | Porto Velho / Rondonia | BR | **KT030821** | **KT030801** | **KT368811** |
| 987 | ROMO 22 | bat | *Pteronotus parnellii* | 2005 | Porto Velho / Rondonia | BR | **KT030822** | - | - |
| 988 | ROMO 23 | bat | *Pteronotus parnellii* | 2005 | Porto Velho / Rondonia | BR | **KT030823** | **KT030804** | - |
| 989 | ROMO 24 | bat | *Pteronotus parnellii* | 2005 | Porto Velho / Rondonia | BR | **KT030824** | - | - |
| 1007 | ROMO 50 | bat | *Pteronotus parnellii* | 2005 | Porto Velho / Rondonia | BR | **KT030825** | - | - |
| 1008 | ROMO 56 | bat | *Pteronotus parnellii* | 2005 | Porto Velho / Rondonia | BR | **KT030826** | - | - |
| 1019 | ROMO 41 | bat | *Pteronotus parnellii* | 2005 | Porto Velho / Rondonia | BR | **KT030827** | - | - |
| 1020 | ROMO 51 | bat | *Pteronotus parnellii* | 2005 | Porto Velho / Rondonia | BR | **KT030828** | - | - |
| 1021 | ROMO 48 | bat | *Pteronotus parnellii* | 2005 | Porto Velho / Rondonia | BR | **KT030829** | - | - |
| 1022 | ROMO 43 | bat | *Pteronotus parnellii* | 2005 | Porto Velho / Rondonia | BR | **KT030830** | **KT030805** | **KT368812** |
| 1023 | ROMO 44 | bat | *Pteronotus parnellii* | 2005 | Porto Velho / Rondonia | BR | **KT030831** | **KT030802** | - |
| 1871 | Ptero 6 | bat | *Pteronotus gymnonotus* | 2009 | Porto Velho / Rondonia | BR | **KT030832** | - | - |
| 1872 | Ptero 11 | bat | *Pteronotus personatus* | 2009 | Porto Velho / Rondonia | BR | **KT030833** | - | - |
| 1873 | Ptero 17 | bat | *Pteronotus personatus* | 2009 | Porto Velho / Rondonia | BR | **KT030834** | **-** | - |
| 1878 | Ptero 8 | bat | *Pteronotus gymnonotus* | 2009 | Porto Velho / Rondonia | BR | **KT030835** | **KT030806** | - |
| **Blood samples of bat infected**  **with *T. wauwau*** | | |  |  |  |  |  |  |  |
| - | PR100 | bat | *Pteronotus gymnonotus* | 2006 | Itabaiana / Sergipe | BR | **KT030836** | - | - |
| - | PR105 | bat | *Pteronotus gymnonotus* | 2006 | Itabaiana / Sergipe | BR | **KT030837** | - | - |
| - | VCT 6227 | bat | *Pteronotus gymnonotus* | 2009 | Parauapebas / Pará | BR | **KT030838** | - | - |
| - | VCT 6236 | bat | *Pteronotus gymnonotus* | 2009 | Parauapebas / Pará | BR | **KT030839** | - | - |
| - | VCT 6238 | bat | *Pteronotus gymnonotus* | 2009 | Parauapebas / Pará | BR | **KT030840** | - | - |
| - | VCT 6239 | bat | *Pteronotus gymnonotus* | 2009 | Parauapebas / Pará | BR | **KT030841** | - | - |
| - | VCT 6254 | bat | *Pteronotus gymnonotus* | 2009 | Parauapebas / Pará | BR | **KT030842** | - | - |
| - | VCT 6379 | bat | *Pteronotus gymnonotus* | 2010 | Parauapebas / Pará | BR | **KT030843** | - | - |
| - | VCT 6409 | bat | *Pteronotus gymnonotus* | 2010 | Parauapebas / Pará | BR | **KT030844** | - | - |
| - | VCT 1103 | bat | *Pteronotus parnellii* | 2007 | Parauapebas / Pará | BR | **KT030845** | - | - |
| - | VCT 3880 | bat | *Pteronotus parnellii* | 2008 | Xinguara / Pará | BR | **KT030846** | - | - |
| - | VCT 4330 | bat | *Pteronotus parnellii* | 2008 | Canaã dos Carajás / Pará | BR | **KT030847** | - | - |
| - | MOL174 | bat | *Pteronotus parnellii* | 2004 | Rio Sono / Tocantins | BR | **KT030848** | - | - |
| - | RB 06 | bat | *Pteronotus parnellii* | 2010 | Ribeirãozinho / Mato Grosso | BR | **KT030849** | - | - |
| - | MN70-07 | bat | *Pteronotus parnellii* | 2010 | São Vicente / Mato Grosso | BR | **KT030850** | - | - |
| - | ROM 97963 | bat | *Pteronotus parnellii* | 1990 | Annai / Upper Takutu-Upper Essequibo | GY | **KT030851** | - | - |
| - | ROM 97965 | bat | *Pteronotus parnellii* | 1990 | Annai / Upper Takutu-Upper Essequibo | GY | **KT030852** | - | - |
| - | ROM 102929 | bat | *Pteronotus parnellii* | 1994 | Surama / Upper Takutu-Upper Essequibo | GY | **KT030853** | - | - |
| - | ROM 102973 | bat | *Pteronotus parnellii* | 1994 | Surama / Upper Takutu-Upper Essequibo | GY | **KT030854** | - | - |
| - | ROM 102990 | bat | *Pteronotus parnellii* | 1994 | Surama / Upper Takutu-Upper Essequibo | GY | **KT030855** | - | - |
| - | ROM 103126 | bat | *Pteronotus parnellii* | 1994 | Surama / Upper Takutu-Upper Essequibo | GY | **KT030856** | - | - |
| - | ROM 103420 | bat | *Pteronotus parnellii* | 1994 | Tropenbos / Upper Demerara-Berbice | GY | **KT030857** | - | - |
| - | ROM 106659 | bat | *Pteronotus parnellii* | 1996 | Upper Takutu-Upper Essequibo | GY | **KT030858** | - | - |
| - | ROM 107348 | bat | *Pteronotus parnellii* | 1997 | Iwokrama Reserve / Potaro-Siparuni | GY | **KT030859** | - | - |
| - | ROM 109024 | bat | *Pteronotus parnellii* | 1997 | Iwokrama Reserve / Potaro-Siparuni | GY | **KT030860** | - | - |
| - | ROM 109292 | bat | *Pteronotus parnellii* | 1997 | Iwokrama Reserve / Potaro-Siparuni | GY | **KT030861** | - | - |
| - | ROM 111534 | bat | *Pteronotus parnellii* | 1999 | Iwokrama Forest / Potaro-Siparuni | GY | **KT030862** | - | - |
| - | ROM 111664 | bat | *Pteronotus parnellii* | 1999 | Iwokrama Forest / Potaro-Siparuni | GY | **KT030863** | - | - |
| - | ROM 111814 | bat | *Pteronotus parnellii* | 1999 | Iwokrama Forest / Potaro-Siparuni | GY | **KT030864** | - | - |
| - | ROM 113739 | bat | *Pteronotus parnellii* | 2001 | Demerara / Mahaica | GY | **KT030865** | - | - |
| - | ROM 113823 | bat | *Pteronotus parnellii* | 2001 | Demerara / Mahaica | GY | **KT030866** | - | - |
| - | ROM 115482 | bat | *Pteronotus parnellii* | 2002 | Essequibo Islands-West Demerara/ Shanklands | GY | **KT030867** | - | - |
| - | ROM 115561 | bat | *Pteronotus parnellii* | 2002 | Essequibo Islands-West Demerara/ Shanklands | GY | **KT030868** | - | - |
| - | ROM 116524 | bat | *Pteronotus parnellii* | 2005 | Kaieteur National Park / Potaro-Siparuni | GY | **KT030869** | - | - |
| - | ROM 116636 | bat | *Pteronotus parnellii* | 2005 | Kaieteur National Park / Potaro-Siparuni | GY | **KT030870** | - | - |
| - | ROM 116651 | bat | *Pteronotus parnellii* | 2005 | Kaieteur National Park / Potaro-Siparuni | GY | **KT030871** | - | - |
| - | ROM 99235 | bat | *Pteronotus parnellii* | 1991 | Petén | GT | **KT030872** | - | - |
| - | ROM 104227 | bat | *Pteronotus parnellii* | 1995 | Parque Nacional Soberania /Canal Zone | PA | **KT030873** | - | - |
| - | ROM 104355 | bat | *Pteronotus parnellii* | 1995 | Parque Nacional Darién / Darien | PA | **KT030874** | - | - |
| - | ROM 104369 | bat | *Pteronotus parnellii* | 1995 | Parque Nacional Darién / Darien | PA | **KT030875** | - | - |
| - | ROM 114151 | bat | *Pteronotus parnellii* | 2002 | Brownsberg Nature Park / Brokopondo | SR | **KT030876** | - | - |
| **Other bat trypanosomes** | |  |  |  |  |  |  |  |  |
| ***T. livingstonei*** | |  |  |  |  |  |  |  |  |
| 1270 |  | bat | *Rhinolophus landeri* | 2006 | Chupanga | MZ | KF192979 | KF192958 | - |
| 1295 |  | bat | *Rhinolophus landeri* | 2006 | Chupanga | MZ | KF192981 | KF192960 | - |
| 1304 |  | bat | *Rhinolophus landeri* | 2006 | Chupanga | MZ | KF192983 | KF192962 | KF192970 |
| 1933 |  | bat | *Rhinolophus landeri* | 2009 | Chupanga | MZ | KF192986 | KF192964 | KF192972 |
| ***T.* sp Panama 1** | |  |  |  |  |  |  |  |  |
| - | 093_AJ_Bohio | bat | *Artibeus jamaicensis* | 2005 | - | PA | KM406889 | - | - |
| - | 134_AJ_Cacao | bat | *Artibeus jamaicensis* | 2005 | - | PA | KM406888 | - | - |
| - | 216_AJ_Guava | bat | *Artibeus jamaicensis* | 2005 | - | PA | KM406898 | - | - |
| - | 278_AJ_Leon | bat | *Artibeus jamaicensis* | 2005 | - | PA | KM406887 | - | - |
| - | 300_AJ_BCI | bat | *Artibeus jamaicensis* | 2005 | - | PA | KM406886 | - | - |
| - | 302_AJ_BCI | bat | *Artibeus jamaicensis* | 2005 | - | PA | KM406884 | - | - |
| - | RNMO56 | bat | *Trachops cirrhosus* | 2012 | Angicos / Rio Grande do Norte | BR | **KT368795** | - | - |
| - | RNMO63 | bat | *Trachops cirrhosus* | 2012 | Angicos / Rio Grande do Norte | BR | **KT368796** | - | - |
| ***T.* sp Panama 2** | |  |  |  |  |  |  |  |  |
| - | 082_AJ_Bohio_2 | bat | *Artibeus jamaicensis* | 2005 | - | PA | KM406907 | - | - |
| - | 092_AJ_Bohio | bat | *Artibeus jamaicensis* | 2005 | - | PA | KM406881 | - | - |
| - | 173_AJ_Gigante | bat | *Artibeus jamaicensis* | 2005 | - | PA | KM406883 | - | - |
| - | 196_AJ_PenaBlanca | bat | *Artibeus jamaicensis* | 2005 | - | PA | KM406882 | - | - |
| - | 275_AJ_Leon | bat | *Artibeus jamaicensis* | 2005 | - | PA | KM406880 | - | - |
| ***T.* sp Panama 3** | |  |  |  |  |  |  |  |  |
| - | 070_AJ_Guanabano | bat | *Artibeus jamaicensis* | 2005 | - | PA | KM406897 | - | - |
| - | 109_AJ_Bohio | bat | *Artibeus jamaicensis* | 2005 | - | PA | KM406879 | - | - |
| - | 121_AJ_Cacao | bat | *Artibeus jamaicensis* | 2005 | - | PA | KM406876 | - | - |
| - | 240_AJ_Leon | bat | *Artibeus jamaicensis* | 2005 | - | PA | KM406875 | - | - |
| - | 268_AJ_Leon | bat | *Artibeus jamaicensis* | 2005 | - | PA | KM406878 | - | - |
| - | 269_AJ_Leon | bat | *Artibeus jamaicensis* | 2005 | - | PA | KM406874 | - | - |
| - | 282_AJ_Leon | bat | *Artibeus jamaicensis* | 2005 | - | PA | KM406877 | - | - |
| - | BACO44 | bat | *Artibeus lituratus* | 2014 | Boyacá | CO | **KT368797** | **KT368800** | - |
| - | BACO46 | bat | *Artibeus lituratus* | 2014 | Boyacá | CO | **KT368798** | **KT368801** | - |
| ***T. vespertilionis*** | |  |  |  |  |  |  |  |  |
|  | P14 | bat | *Pipistrellus pipistrellus* | 1972 | - | UK | AJ009166 | AJ620283 | AF116564 |
| ***T.* sp bat** | |  |  |  |  |  |  |  |  |
| 60 |  | bat | *Rousettus aegyptiacus* | 1997 | - | GA | AJ012418 | GQ140365 | KF192977 |
| ***T. rangeli*** | |  |  |  |  |  |  |  |  |
| 643 |  | bat | *Platyrrhinus lineatus* | 2003 | Mato Grosso do Sul | BR | FJ900242 | GQ140364 | EU867800 |
| 1719 |  | bat | *Artibeus planirostris* | 2005 | Mato Grosso do Sul | BR | EU867813 | **KT368802** | EU867799 |
| ***T. dionisii*** | |  |  |  |  |  |  |  |  |
| 211 |  | bat | *Eptesicus brasiliensis* | 2000 | São Paulo | BR | FJ001666 | GQ140362 | - |
| 495 |  | bat | *Carollia perspicillata* | 2002 | Amazonas | BR | FJ001667 | GQ140363 | - |
| - | P3 | bat | *Pipistrellus pipistrellus* | 1971 | - | UK | AJ009151 | AJ620271 | - |
| - | PJ | bat | *Pipistrellus pipistrellus* | 1971 | - | BE | AJ009152 | - | - |
| - | x842 |  | *Nyctalus noctula* | 2006 | - | UK | FN599058 | FN599055 | - |
| ***T. erneyi*** | |  |  |  |  |  |  |  |  |
| 1293 |  | bat | *Tadarida sp.* | 2006 | Chupanga | MZ | JN040987 | JN040964 | - |
| 1294 |  | bat | *Tadarida sp.* | 2006 | Chupanga | MZ | JN040988 | JN040965 | - |
| 1946 |  | bat | *Mopys condylurus* | 2009 | Chupanga | MZ | JN040989 | JN040969 | - |
| ***T. c. marinkellei*** | |  |  |  |  |  |  |  |  |
| - | B7 | bat | *Phyllostomus discolor* | 1974 | Bahia | BR | AJ009150 | AJ620270 | - |
| 344 |  | bat | *Carollia perspicillata* | 2001 | Monte Negro/ Rondonia | BR | FJ001664 | GQ140360 | - |
| 501 |  | bat | *Carollia perspicillata* | 2002 | Porto Velho/Rondonia | BR | FJ001665 | GQ140361 | - |
| ***T. cruzi*** | |  |  |  |  |  |  |  |  |
| 1122 |  | bat | *Myotis albescens* | 2004 | São Paulo | BR | FJ001628 | GQ140359 | - |
| 1994 |  | bat | *Myotis levis* | 2004 | São Paulo | BR | FJ900241 | GQ140358 | - |
| 417 |  | bat | *Thyroptera tricolor* | 2002 | Amazonas | BR | AF900240 | - | - |
| 507 |  | bat | *Carollia perspicillata* | 2002 | Amazonas | BR | FJ900240 | GQ140352 | - |
| **Trypanosomes of other mammals** | | |  |  |  |  |  |  |  |
| - | HochNdi1 | monkey | *Cercopithecus nictitans* | 2004 | - | CM | FM202493 | FM164794 | - |
| - | NanDoum1 | palm civet | *Nandinia binotata* | 2004 | - | CM | FM202492 | FM164793 | - |
| - | H25 | kangaroo | *Macropus giganteus* | 1997 | - | AU | AJ009168 | AJ620276 | **KT368809** |
| - | G8 | **woylie** | **Bettongia penicillata** | 2013 | - | AU | KC753537 | KC812988 | - |
| - | BDA1 | woylie | *Bettongia lesueur* | 2009 | - | AU | FJ823108 | - | - |
| - | D15 | possum | *Trichosurus vulpecula* | 2009 | - | AU | JN315381 | JN315395 | - |
| - | D17 | possum | *Trichosurus vulpecula* | 2009 | - | AU | JN315382 | JN315396 | - |
| - | D64 | possum | *Trichosurus vulpecula* | 2009 | - | AU | JN315383 | JN315397 | - |
| - | BRA2 | rodent | *Rattus fuscipes* | 2007 | - | AU | FJ823117 | - | - |
| ***T. conorhini*** | |  |  |  |  |  |  |  |  |
| 25e |  | rodent | *Rattus rattus* | 1947 | - | BR | AJ012411 | AJ620267 | AJ272600 |
| ***T. rangeli*** | |  |  |  |  |  |  |  |  |
| - | RGB | dog | *Canis familiaris* | 1949 | - | CO | AJ009160 | AF053742 | AJ012419 |
| 86 | AM80 | human | *Homo sapiens* | 1996 | Amazonas | BR | AY491766 | JN040973 | - |
| 23 | SC58 | rodent | *Echimys dasythrix* | - | Santa Catarina | BR | AY230233 | **KT368804** | - |
| 14 | PG | human | *Homo sapiens* | - | Panama | PA | AJ012416 | **KT368805** | - |
| 31 | San Agustin | human | *Homo sapiens* | - | - | CO | AJ012417 | **KT368806** | - |
| 020 | Macias | human | *Homo sapiens* | - | - | VE | AJ012415 | - | - |
| 261 | - | human | *Homo sapiens* | *-* | Rio Negro/ Amazonas | BR | AY491758 | **KT368807** | - |
| 328 | 1625 | human | *Homo sapiens* | - | - | SV | AY491738 | **KT368808** | - |
| 900 | - | triatomine | *Rhodnius pictipes* | *-* | Manaus/ Amazonas | BR | **KT368799** | **KT368803** | - |
| ***T. lewisi*** | |  |  |  |  |  |  |  |  |
| - | Molteno B3 | rodent | *Rattus rattus* | - | - | UK | AJ009156 | AJ620272 | - |
| ***T. microti*** | |  |  |  |  |  |  |  |  |
|  | TRL 132 | vole | *Microtis agrestis* | - | - | UK | AJ009158 | AJ620273 | - |
| ***T. cruzi*** | |  |  |  |  |  |  |  |  |
| 30 | G | opossum | *Didelphis marsupialis* | 1983 | Amazonas | BR | AF239981 | GQ140351 | - |
| 34 | Y | human | *Homo sapiens* | 1953 | São Paulo | BR | AF301912 | GQ140353 | - |
| 845 | MT3663 | triatomine | Panstrongylus geniculatus | - | Amazonas | BR | AF288660 | JN040971 | - |
| 844 | MT3869 | human | Homo sapiens | - | Amazonas | BR | AF303660 | GQ140355 | - |

^a^ Cryopreserved cltures of bat trypanosomes bat blood samples deposited in the Trypanosomatid Culture Collection (TCC-USP) of the Department of Parasitology, University of São Paulo, São Paulo, Brazil. TCC correspond to codes of cultures deposited in this collection. ^b^ GenBank accession number of gene sequences determined in this study are indicated in bold. BR, Brazil; GY, Guyana; GT, Guatemala; SR, Suriname; PA, Panamá; MZ, Mozambique; CO, Colombia; UK, United Kingdom; GA, Gabon; BE, Belgium; CM, Cameroon; AU, Australia; VE, Venezuela; SV, El Salvador
